# Investigating the therapeutic mechanism of Xiaotan Sanjie Formula for gastric cancer via network pharmacology and molecular docking: A review

**DOI:** 10.1097/MD.0000000000035986

**Published:** 2023-11-17

**Authors:** Xinxin Xu, Zhihong Yu, Shuying Zeng

**Affiliations:** a The Second Clinical Medical College, Zhejiang Chinese Medicine University, Hangzhou, China; b Cancer Institute of Integrated Tradition Chinese and Western Medicine, Zhejiang Academy of Traditional Chinese Medicine, Tongde Hospital of Zhejiang Province, Hangzhou, China.

**Keywords:** gastric cancer, molecular docking, network pharmacology, XTSJ Formula

## Abstract

Xiaotan Sanjie Formula (XTSJF), a traditional Chinese prescription, holds promising potential in addressing gastric cancer (GC). Despite this, the fundamental constituents and underlying mechanisms that define XTSJF’s attributes remain enigmatic. Against this backdrop, this study endeavors to unravel the latent mechanisms driving XTSJF’s impact on GC, leveraging the synergistic prowess of network pharmacology and molecular docking methodologies. To understand the potential mechanism of XTSJF against GC, this study used network pharmacology, molecular docking, and bioinformatics analytic methodologies. There are 135 active components where the active ingredients with a higher degree value are quercetin, β-sitosterol, naringenin, nobiletin, and kaempferol and 167 intersecting targets in which TP53, MAPK3, MAPK1, STAT3, and AKT1 were key targets were identified in XTSJF in the treatment of GC. According to GO and KEGG analyses, XTSJF is mostly involved in the positive control of transcription from the RNA polymerase II promoter, enzyme interaction, and other biological processes in GC. KEGG analysis shows that XTSJF treated GC primarily by regulating signaling pathways including the TNF, PI3K-Akt, and MAPK signaling pathways. According to the results of the PPI network and molecular docking, quercetin, β-sitosterol, naringenin, nobiletin, and kaempferol exhibit stronger affinity with TP53, MAPK3, MAPK1, STAT3, and AKT1. This study indicates the active components of XTSJF as well as its possible molecular mechanism against GC, and it serves as a foundation for future fundamental research.

## 1. Introduction

Gastric cancer, a highly malignant neoplasm, stands prominent among the global top 5 malignancies in terms of both incidence and mortality rates.^[1]^ The clinical management of this malignancy has faced substantial challenges due to its marked heterogeneity and robust biological aggressiveness. This intricate nature of gastric cancer (GC) has contributed to the gradual pace of advancements in its clinical treatment. Consequently, an imminent requirement arises for novel antitumor therapeutics characterized by heightened efficacy and reduced toxicity.

The pressing clinical demand for innovative antitumor agents and efficacious treatment modalities, capable of markedly enhancing patient prognosis, is incontrovertible. In this context, Traditional Chinese Medicine (TCM) emerges as a historical cornerstone in the realm of malignant disease prevention and treatment. Furthermore, TCM assumes a pivotal role as a complementary and alternative strategy within the comprehensive treatment of GC. Notably, TCM brings about significant improvements in the overall quality of life^[2]^ and effectively boosts the survival rates among patients grappling with GC.^[3,4]^ The intricate clinical challenges posed by GC necessitate fresh perspectives in treatment. Traditional Chinese Medicine, with its historical underpinnings and demonstrated clinical efficacy, presents itself as a compelling complement to conventional therapeutic regimens. By amalgamating the strengths of both TCM and modern medicine, a promising synergy emerges, offering a renewed outlook for more effective, safer, and holistic approaches toward addressing the global burden of GC.

Xiaotan Sanjie Formula (XTSJF), an empirical formula rooted in the “phlegm” theory, has exhibited a steadfast presence in the therapeutic landscape of GC for over 3 decades. The formula’s application in GC prevention and treatment has been extensively documented. Early clinical investigations have unveiled XTSJF’s capability to significantly ameliorate card scores in patients afflicted with advanced gastrointestinal tumors, thereby conferring an extension in survival time.^[2,5,6]^ Complementary experimental inquiries have shed light on the plausible mechanisms underpinning XTSJF’s anti-gastric cancer effects, encompassing impediment of metastasis, promotion of apoptosis through anti-angiogenic pathways, and restraint of epithelial-mesenchymal transition, among other facets.^[7,8]^

The intricacies intrinsic to GC treatment stem from the formidable spatiotemporal heterogeneity exhibited by the disease, the intricate tumor microenvironment milieu, and the scarcity of prominent mutational targets. In contrast, XTSJF embodies a composite medicinal amalgamation boasting manifold targets and mechanisms, thereby presenting a plausible avenue to surmount the prevailing challenges inherent in GC therapy. Nevertheless, extant studies have faltered in presenting a comprehensive multi-tiered explication of XTSJF’s therapeutic and disease-modulating actions within the context of GC treatment.

This review aims to bridge the existing knowledge gap by unraveling the intricate tapestry of XTSJF’s therapeutic potential against gastric malignancies. Through rigorous exploration of its pharmacological attributes and mechanistic engagements, this study seeks to furnish a holistic comprehension of XTSJF’s utility within the domain of GC treatment. By synthesizing empirical wisdom with mechanistic elucidation, a more nuanced understanding of XTSJF’s applicability in addressing the manifold challenges posed by GC is envisaged.

Network pharmacology, heralding a novel paradigm in Chinese medicine research, orchestrates a “drug-target” nexus by amalgamating drug or prescription attributes with potency, thereby effectuating target prediction and mechanism delineation.^[9]^ The realm of TCM benefits substantially from network pharmacology, with its focal points converging on the aggregation of TCM’s chemical constituents via databases and the application of computational biology to unravel potential component-target associations. The synthesis of a “herbal-component-target-signaling pathway” network culminates from these pursuits, furnishing preliminary insights into the intricate nexus of TCM’s multifarious components, targets, and pathways. This engenders a nuanced comprehension of TCM’s pharmacological underpinnings, facilitating the mastery of its molecular mechanisms. Notably, the confluence of this theory parallels TCM’s cardinal principle of symptom differentiation and treatment, reinforcing its congruence with network pharmacology. As current TCM investigations progressively assimilate network pharmacology methodologies, a corpus of studies converges to proffer foundational and empirical elucidations for diverse TCM conundrums. In this context, this study undertakes a comprehensive network pharmacology exploration to dissect the principal components and molecular biological mechanisms intrinsic to XTSJF in the context of GC. This endeavor strives to furnish pertinent referential resources for subsequent investigations, while concurrently exemplifying the symbiotic synergy between network pharmacology and TCM research.

## 2. Resources and procedures

### 2.1. Target component selection for XTSJF

The compounds and their respective target interactions within XTSJF, comprising Pinellia ternate, Rhizoma Arisaematis, Poria, Fructus aurantii immatures, citrus, Bulbus Fritillariae Cirrhosae, Semen Sinapis albae, and Glycyrrhiza uralensis, were gleaned from the TCMSP database (accessible at https://old.tcmsp-e.com/tcmsp.php).^[10]^ Additionally, other active chemical components (Endothelium corneum, centipede, and scorpion), not traceable within TCMSP, were procured from the SymMap database. Oral bioavailability (OB) quantifies the extent to which orally ingested medicine enters the bloodstream, while drug similarity (DL) gauges the likeness between a chemical and established drugs.^[10,11]^ Employing criteria of OB ≥ 30% and DL ≥ 0.18, we ascertained the principal components of XTSJF. Subsequently, we harnessed the resources of PubChem, a repository for organic small molecule bioactivity information, to extract primary chemical components housed within XTSJF’s central molecular structures. Employing pharmacokinetic data retrieval filters set at probability ≥ 0.7, we discerned potential component targets through SwissTargetPrediction (accessible at http://SwissTargetPrediction.ch/). The ensuing identified protein targets were then harmonized into standardized gene names, leveraging the UniProt database.

### 2.2. Establishing the network of herb-compound-target interactions

Utilizing the Cytoscape 3.9.1 tool, a platform renowned for its capacity to portray intricate biological pathways and molecular associations,^[12]^ we constructed the “herb-compound-target” network. This network was formed by amalgamating all potential XTSJF targets and corresponding chemical compounds. In this visually informative representation, each component’s interaction with its respective target is visually conveyed through connecting lines, with nodes symbolizing individual components or targets within the herb-compound-target network.

### 2.3. Acquisition of targets of GC

Looking up target genes linked to GC using the keywords “gastric cancer” in the public databases OMIM^[13]^ and GeneCards.^[14]^ The relationships between targets and diseases in the GeneCards database are correlated with scores, so filter indicators based on scores: the higher the score, the stronger the relevance of the target to the disease. Removing the duplicate targets, the principal targets of GC were obtained.

### 2.4. Acquisition of relevant targets and Venn diagram

We plotted Venn diagrams to identify the intersection of targets of XTSJF and GC, where the intersection is a valid target for the XTSJF in the treatment of GC.

### 2.5. Construction of a component-target network of XTSJF in GC

For visualization of the relationship of XTSJF in the treatment of GC, potential active components, and main protein targets of XTSJF in GC were incorporated into Cytoscape3.9.

### 2.6. Construction of protein targets network and enrichment analysis targets function and pathway

To unravel the protein-protein interactions (PPI) germane to XTSJF’s role in GC’s pathophysiology, we harnessed the STRING database. The overlapping set of targets was fed into this database, utilizing filtering criteria encompassing the “Homo sapiens” organism and a stringent confidence level (0.9). This yielded a repertoire of targeted proteins that formed the basis for constructing a PPI network in Cytoscape 3.9.1. This software not only facilitates network visualization but also computes pivotal network metrics including degree, betweenness centrality, closeness centrality (CC), and average shortest path length. Employing a meticulous approach, we scrutinized these network metrics, favoring target nodes with degree values, betweenness centrality, and CC surpassing the median values within the PPI network. This methodology enabled the identification of 10 core targets for future investigations. In a bid to unravel the biological context of putative GC targets, the DAVID database came into play, allowing the aggregation of valuable Gene Ontology (GO) analysis and Kyoto Encyclopedia of Genes and Genomes (KEGG) data. The outcomes of these analyses were artfully presented through histograms and bubble plots, each category thoughtfully sequenced in order of significance. The spectrum encompassed cellular components, molecular functions, and biological processes, all of which underwent meticulous screening via GO analysis. Additionally, KEGG enrichment analysis unearthed crucial signaling pathways embedded within pivotal biological processes.

### 2.7. Molecular docking

#### 2.7.1. Protein receptor file preparation.

Search for the target proteins in the RCSB PDB (https://www.rcsb.org/) and save them as PDB format files based on the results of the prior network pharmacology screening. Following that, the protein was configured in PyMOL2.5 to optimize the protein-ligand interactions: Remove additional molecules that were bonded to the target protein structures, as well as any water, organic, and other compounds. The target proteins were then loaded into AutoDockTools1.5.7 to perform hydrogen addition, charge calculations, and receptor designation. The results were then saved in PDBQT format.

#### 2.7.2. Drug receptor file preparation.

The primary component of XTSJF has been identified based on prior research, then download the drug molecule structures as an SDF format file from the PubChem database. The molecule structures in sdf format were converted to mol2 formats using Chem3D. In AutoDockTools1.5.7, we also detect the root and configure the torsion tree. Exporting to a ligand file in PDBQT format is the last step.

#### 2.7.3. Molecular docking and visual representation.

After importing the receptor and ligand PDBQT structures, the molecular docking parameters were defined within AutoDockTools 1.5.7. Subsequently, the docking process was executed in AutoDock 4, entailing the identification of protein macromolecules, integration of small drug molecules, configuration of operational protocols, and specification of docking parameters. The minimal binding energy was computed in the PDBQT format. To conclude, the resultant composite in PDBQT format was converted into PDB format using the OpenBabelGUI software. For the final visual representation, the composite PDB format file was imported into PyMol 2.5.

## 3. Results

### 3.1. The study of network pharmacology

#### 3.1.1. Screening for XTSJF’s major active components and targets.

With the screening conditions as OB ≥ 30% and DL ≥ 0.18 in TCMSP and probability ≥ 0.7 in SwissTargetPrediction, a total of 135 active compounds and 2360 main targets were obtained.

#### 3.1.2. Construction herb-compound-target network of XTSJF.

**Figure 1.**
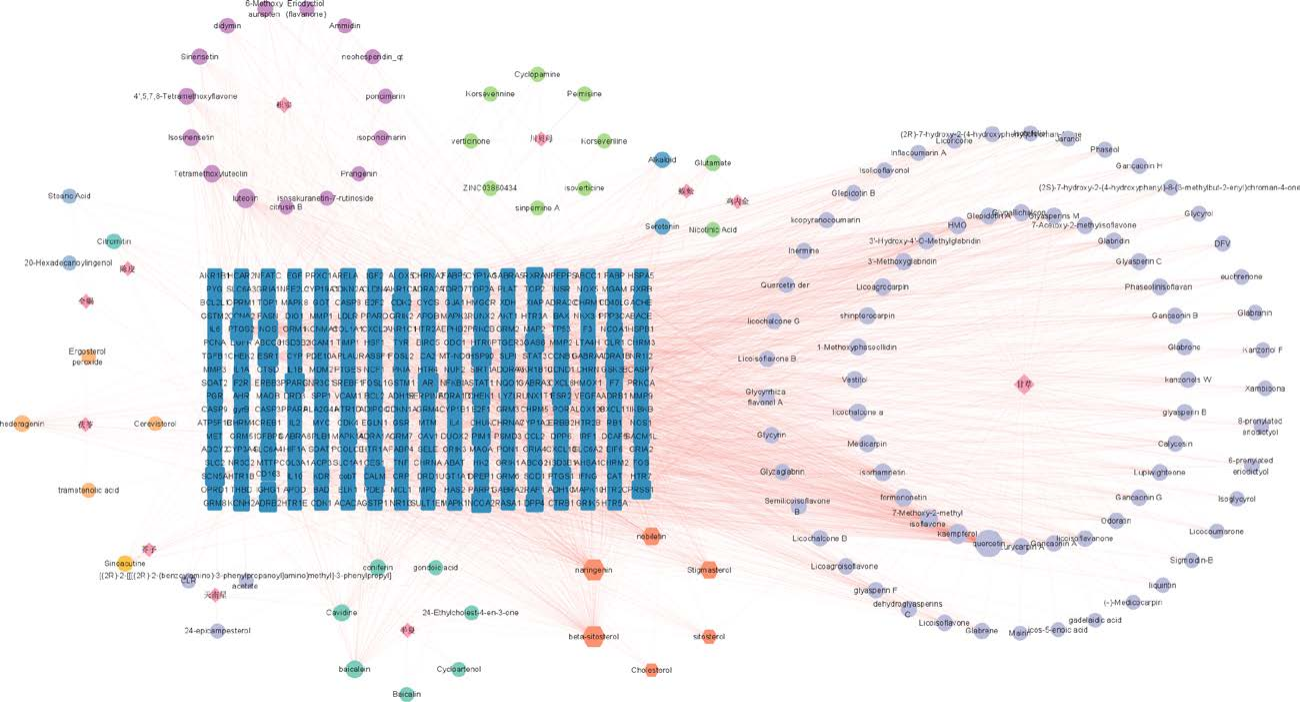
Herb-compound-target network of XTSJF. (The diamonds represent the XTSJF components, 73 purple circles represent the Glycyrrhiza uralensis component, 1 yellow circle represents the Semen Sinapis albae component, 4 yellowish circles represent the Poria component, 2 blue circles represent the scorpion component, 1 light green circle represents the citrus component, 15 dark purple circles representing the Fructus aurantii immaturus component, 8 light green circles representing the Bulbus Fritillariae Cirrhosae component, 2 dark blue circles representing the centipede component, 2 light green circles representing the Endothelium corneum component, 3 light purple circles represent the Rhizoma Arisaematis component, 7 green circles represent the Pinellia ternat component, and 6 orange diamonds represent the shared components in the 11 XTSJF herbs; the blue rectangles represent the potential targets of XTSJF). XTSJF = Xiaotan Sanjie Formula.

Based on the multi-component and multi-target properties of TCM compound prescriptions, we constructed the herb-component-target network. According to Figure 1, there are 440 nodes and 2434 edges.

#### 3.1.3. Identification of XTSJF targets for GC treatment.

Gastric cancer targets were retrieved from GeneCards and OMIM. With a relevance score of ≥ 10 for GeneCards and the subject terms “gastric cancer” and “gastric malignancy” for OMIM, combining all the results and removing duplicates. In total, 2074 intersections are obtained.

#### 3.1.4. Venn diagram drawing.

**Figure 2.**
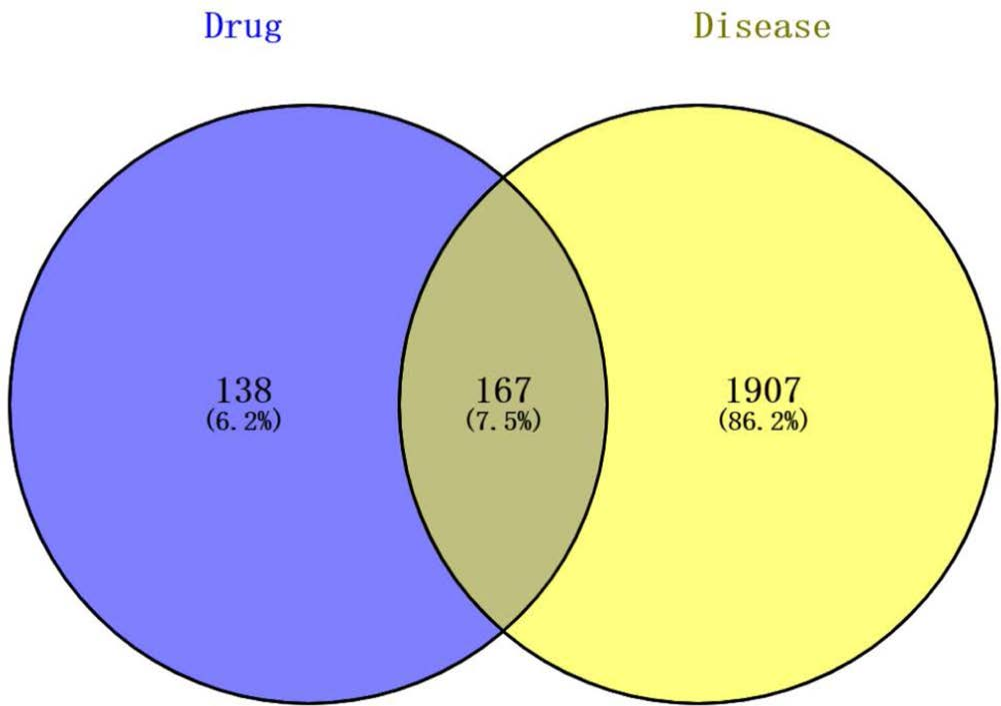
The target of XTSJF and the target of gastric cancer are depicted in a Venn diagram. XTSJF = Xiaotan Sanjie Formula.

We imported the probable ingredient targets and sensis targets of XTSJF utilizing the online Venn diagram tool (Venny 2.1.0) to gather the overlapping targets of medications and disorders. In total, there are 167 intersecting targets, as shown in Figure 2.

#### 3.1.5. Component-target network analysis.

**Figure 3.**
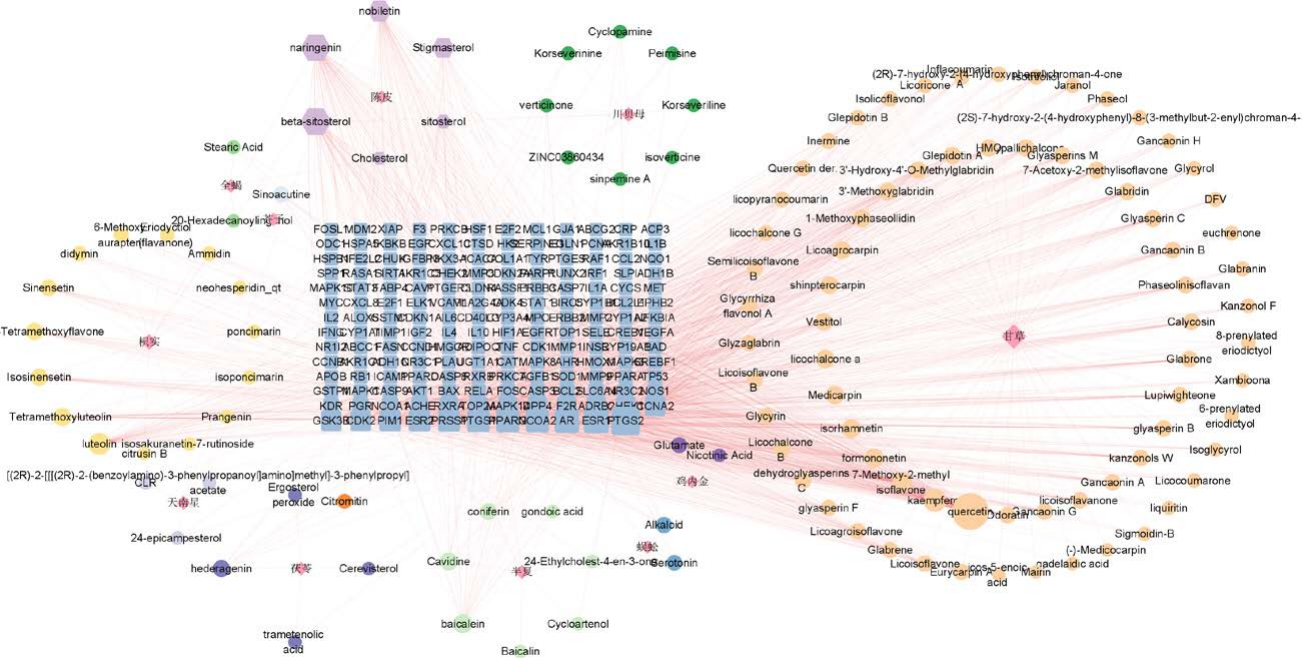
XTSJF’s active component-effective target network diagram (The diamond represents the elements that make up XTSJF, with orange standing in for the licorice element, dark purple for the keratin element, blue for the centipede element, green for the hemipterocarpus element, purple for the poria element, gray-purple for the chrysanthemum element, yellow for the honeysuckle element, light blue for the hyssop element, light green for the scorpion element, and light). XTSJF = Xiaotan Sanjie Formula.

After database prediction and removal of duplicate values, the 2 targets, XTSJF and GC, were combined to obtain a total of 167 common targets. A “component-target” network of XTSJF in GC was also constructed (Fig. 3), in which quercetin, β-sitosterol, naringenin, nobiletin, kaempferol, and luteolin were considered to be the main active components of XTSJF in GC.

#### 3.1.6. Construction of PPI network.

**Figure 4.**
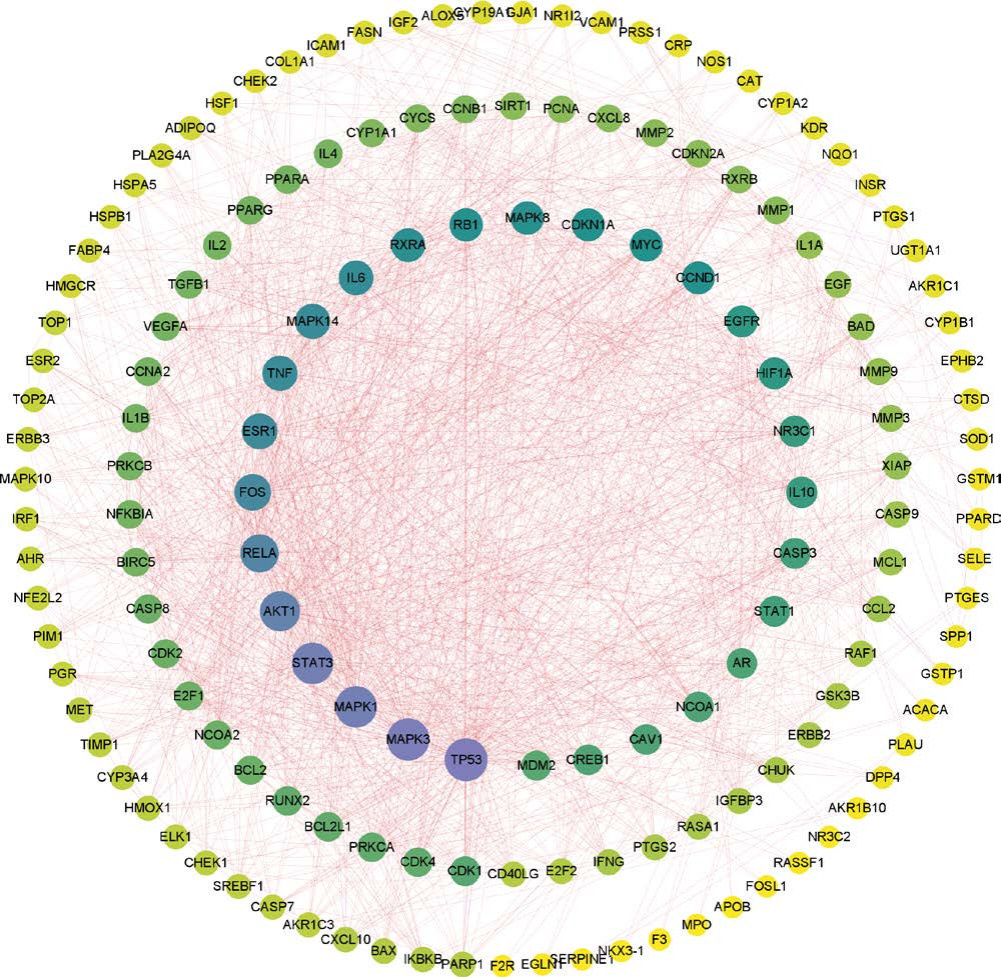
XTSJF’s PPI network and targets for stomach cancer. Proteins are represented by nodes, and the degree of protein binding is indicated by the color yellow. The protein-protein relationship is shown by the edge. XTSJF = Xiaotan Sanjie Formula, PPI = the protein-protein interactions.

Uploading the intersection target to the STRING database with a confidence level of 0.9 sets, and a PPI network graph of the target was got. We imported the data into Cytoscape 3.9.1 to plot the protein network graph, which comprised 153 nodes and 1652 edges. The corresponding degree values increase with the size of the nodes. Network locations were determined based on the degree values. As shown in Figure 4, targets in the center of the network include TP53, MAPK3, MAPK1, STAT3, and AKT1, among others.

#### 3.1.7. Results of the target function and pathway enrichment analysis.

**Figure 5.**
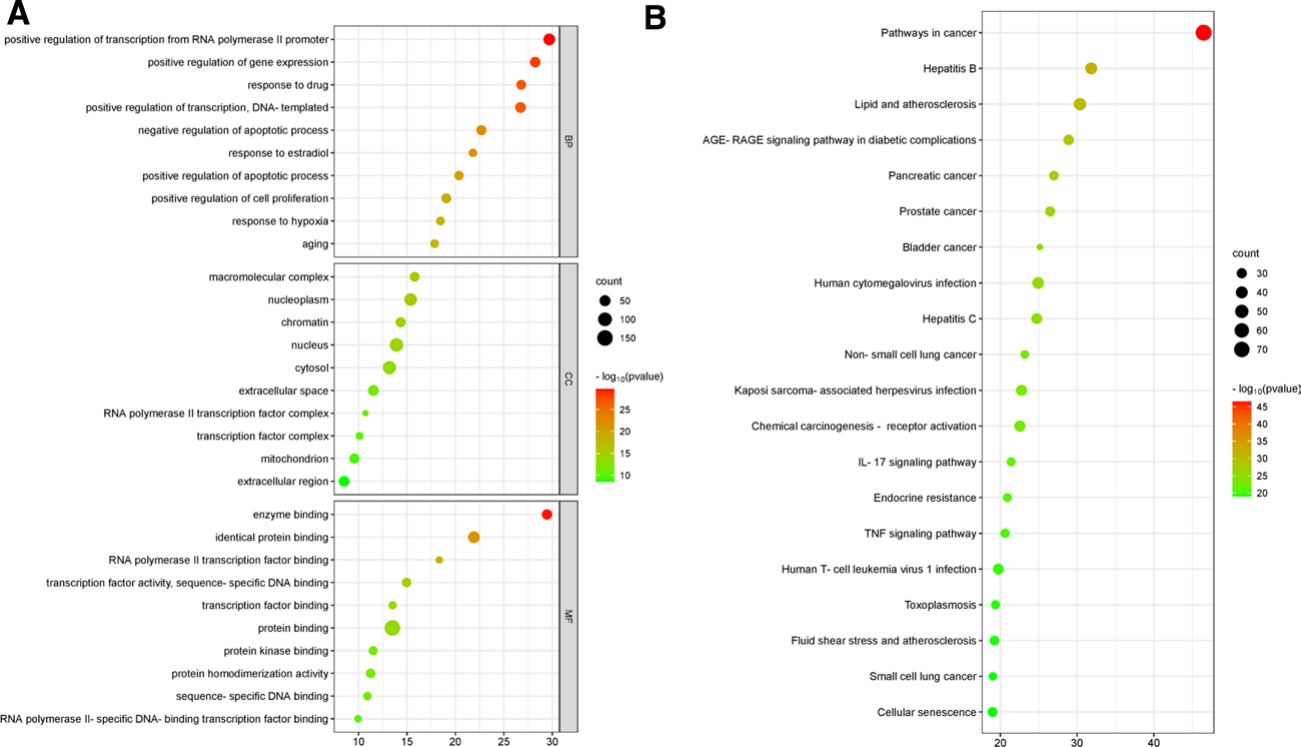
Gene target protein analysis for XTSJF using GO and KEGG. (a) Genes implicated in GO-CC, GO-BP, and GO-MF analyses were discovered by GO enrichment analysis. (b) Bioinformatics data-based KEGG pathway study of the molecular signaling route for GC therapy with XTSJF phacoemulsification. BP = biological process, CC = cellular component, GC = gastric cancer, GO = Gene Ontology, KEGG = Kyoto Encyclopedia of Genes and Genomes, MF = molecular function, XTSJF = Xiaotan Sanjie Formula.

Under the screening condition of *P* < .05, effective targets were annotated using the DAVID database, and 1126 biological process (BP) items were found. These items were primarily connected to transcription from the RNA polymerase II promoter, gene expression, drug response, apoptotic process, estradiol response, and other topics. There were 68 different cellular components (CC) items, most of which were connected to the macromolecular complex, nucleoplasm, chromatin, and nucleus. There were 139 entries for molecular functions (MF), mostly relating to the interaction of enzymes, proteins with similar structures, RNA polymerase II transcription, etc. We respectively select the top 10 results to draw a bubble chart among BP, CC, and MF. These results are shown in Figure 5A. 388 KEGG pathways were enriched under the condition of *P* < .05, mainly including the MAPK signaling pathway, PI3K-AKT signaling pathway, tumor necrosis factor (TNF) signaling pathway, etc. Select the top 20 pathways to draw a bubble chart, and measure the enrichment degree of KEGG by the number of core targets enriched to this pathway and LG *P* value, as shown in Figure 5B.

#### 3.1.8. Molecular docking.

**Figure 6.**
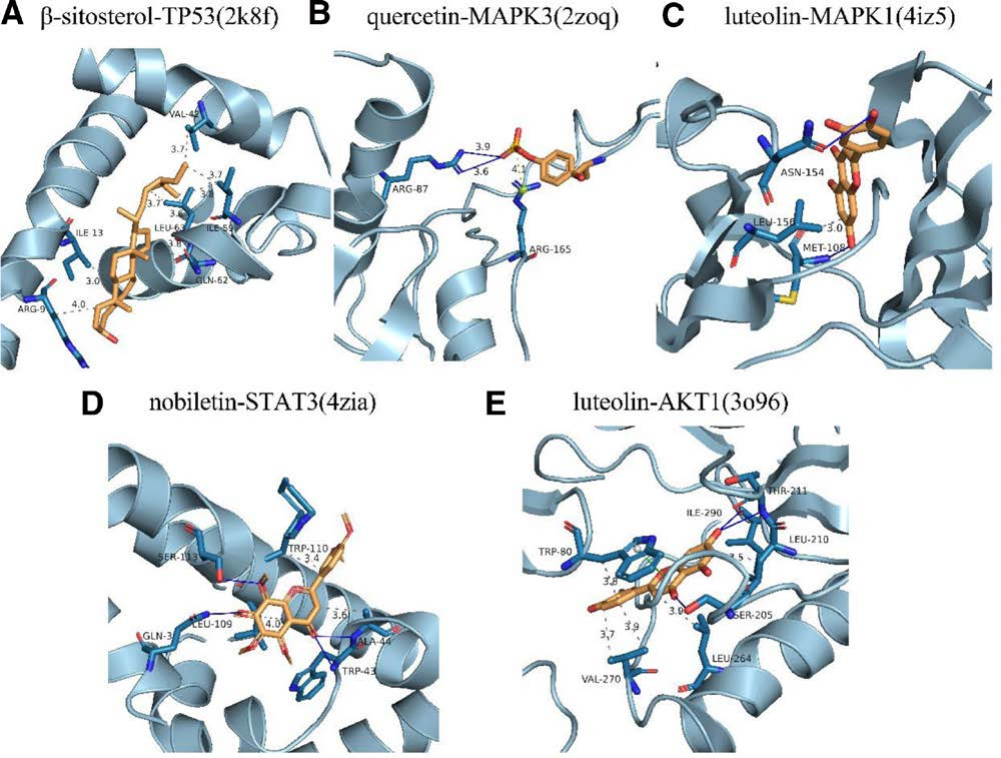
Results of the main chemical components of the XTSJF’s active molecules docking. The blue bar model shows the interactions between the proteins (A) TP53 (2K8F) with the β-sitosterol molecule, (B) MAPK3 (2ZOQ) with the Quercetin molecule, (C) MAPK1 (4IZ5) with the Luteolin molecule, (D) STAT3 (4ZIA) with the Nobiletin molecule and (E) AKT1 (3O96) with the Luteolin molecule. Remainders at the binding site are shown as lines. Dark dashed lines signify p-p interactions, while light dashed lines signify hydrogen bonds. XTSJF = Xiaotan Sanjie Formula.

The primary ingredients of the XTSJF (quercetin, beta-sitosterol, naringenin, nobiletin, kaempferol, and luteolin) were obtained by topological study and calculations. Using AutoDockTools1.5.7, the possible targets and active ingredients of the XTSJF in GC were docked. It was hypothesized that there would be a higher likelihood of pharmacological activity if the conformation of the ligand and receptor binding was more stable. TP53, MAPK3, MAPK1, STAT3, and AKT1 were identified as the top 5 main targets based on degree values (Table 1). The components were molecularly docked using AutoDockTools1.5.7, and the results between ligand small molecules and receptor proteins were as follows: A docking binding energy of less than −5.0 kcal/mol indicates good binding activity, and less than −7.0 kcal/mol implies high binding activity.^[15,16]^ With binding energies of below −7.0 kcal/mol, all 5 of the primary ingredients demonstrated an exceptionally high binding affinity for MAPK3, showing the drug’s potent affinity for the target. The particular docking outcomes are displayed in Figure 6.

**Table 1:** Docking results of the target protein and active compound.

Components	Docking binding energies/(KJ·mol^−1^)				
	TP53 (2k8f)	MAPK3 (2zoq)	MAPK1 (4iz5)	STAT3 (4zia)	AKT1 (3o96)
Quercetin	−6.3	−9	−7.4	−6.9	−9.6
β-sitosterol	−7.3	−7.1	−7.1	−7.1	−6.8
Naringenin	−6.5	−8.7	−7.5	−7	−9.5
Nobiletin	−6.5	−6.5	−6.8	−7.4	−9
Kaempferol	−6.4	−8.9	−7.1	−7.1	−8.6
Luteolin	−6.6	−9	−7.7	−7.2	−9.8

### 3.2. Bioinformatics analysis

**Figure 7.**
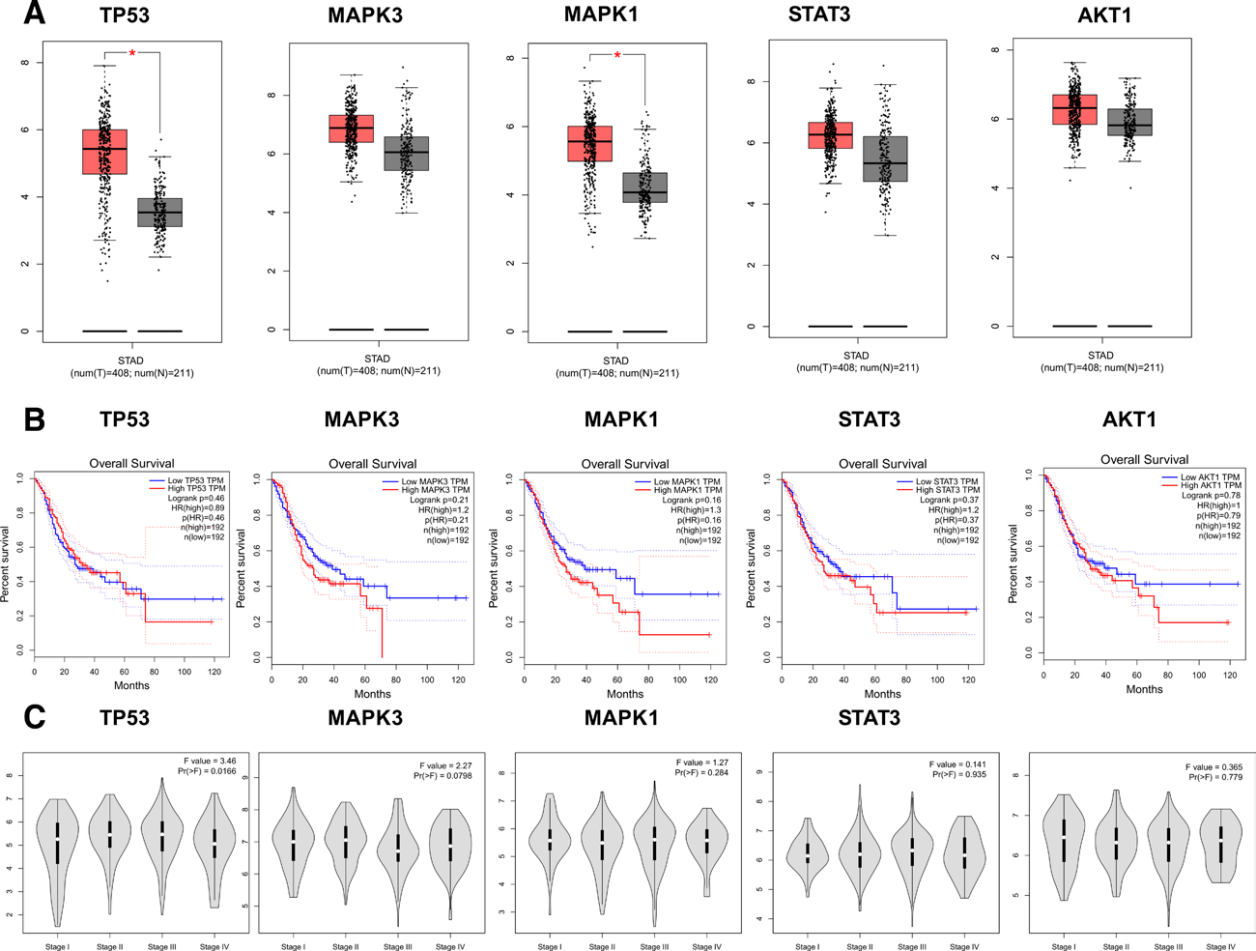
OS, pathogenic stage, and mRNA expression level in the GEPIA database. (A) Box plots displaying the amounts of TP53, MAPK3, MAPK1, STAT3, and ATK1 mRNA expression. Gray denotes normal, whereas red denotes tumor. (B) The line graphs in GEPIA display the OS of the hub genes. Patients with high (red) and low (blue) GC expressions are compared on the survival curve. (C) The violin diagram in the GEPIA database displays the stage plot of mRNA expression level and disease stage. GC = gastric cancer.

**Figure 8.**
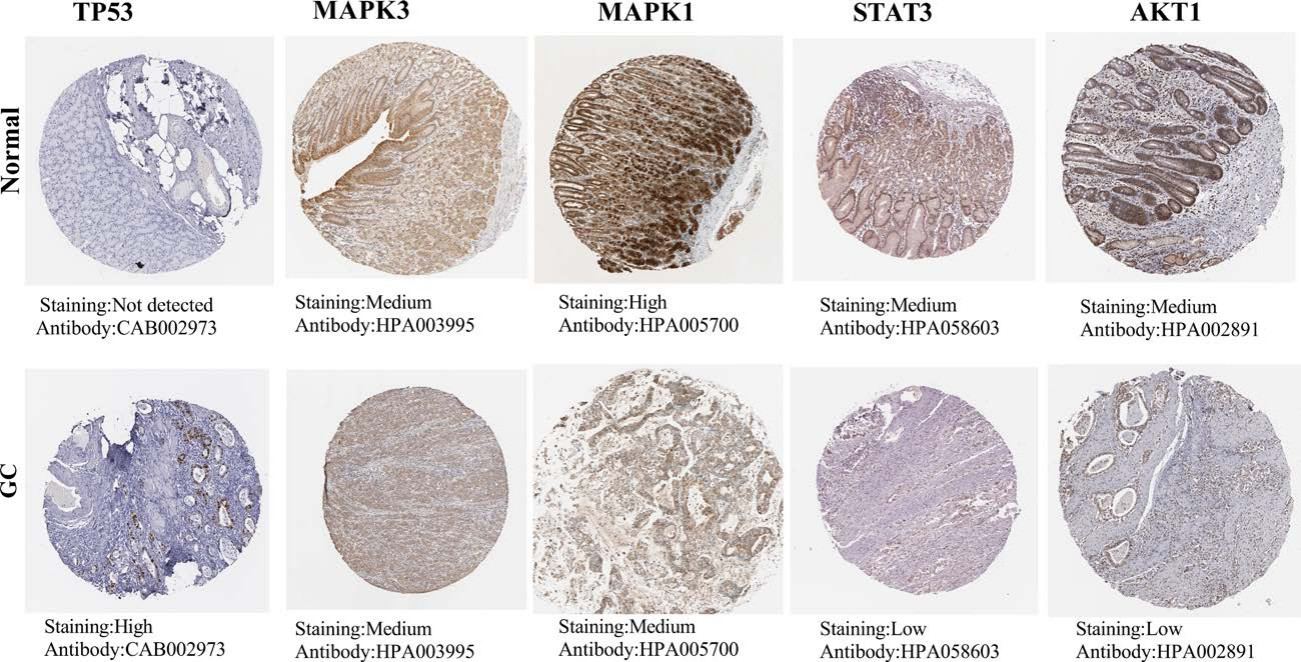
The HPA database’s protein expression levels.

**Figure 9.**
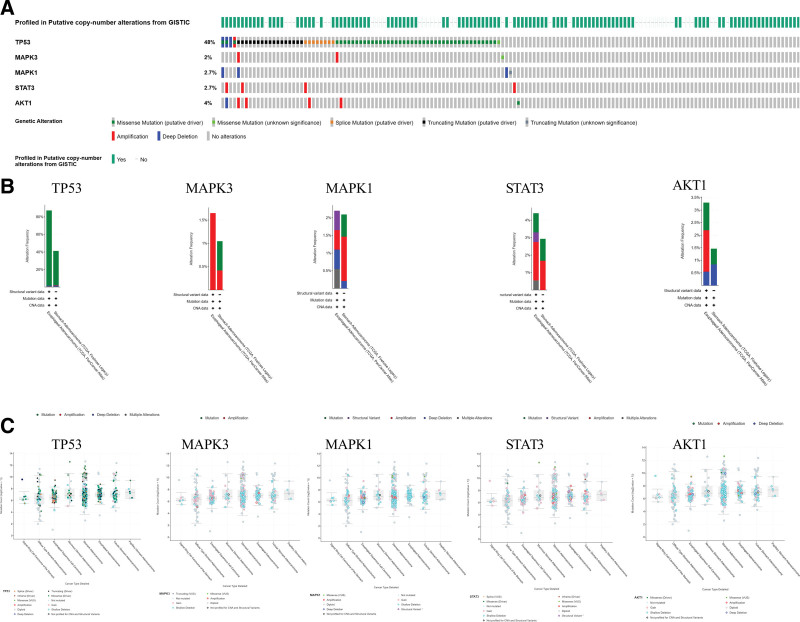
Genetic information of hub targets. (A) Data from TCGA of gastric adenocarcinoma showed that 376 of 660 patients (57%) had genetic mutations in these 5 targets. (B) The diagram shows the genetic variation of 5 targets. (C) The diagram shows the number of gene mutations in different types of gastric cancer.

Statistics from the GEPIA database showed that TP53 and MAPK1 had very high levels of mRNA expression in GC tissues (Fig. 7A). The relationship between the mRNA levels of the pivotal targets and the pathological stage of GC reveals that the levels of TP53 changed significantly with the pathological stage (Fig. 7C), even though survival analysis of the pivotal targets did not reveal any differences between these targets and survival prognosis (*P* > .05, Fig. 7B). Results from the HPA database showed that in healthy stomach tissues, the critical targets were expressed differentially. TP53 was expressed at higher levels in GC tissues compared to healthy gastric tissues, but MAPK1, STAT3, and AKT1 expression were at lower levels (Fig. 8). 376 out of 660 patients (or 57%) had genetic mutations in these 5 targets, according to the cBioPortal database (Fig. 9A). Five targets’ genetic variation, in general, was also examined (Fig. 9B). The amount of gene mutations in various types of GC is depicted in Figure 9C.

## 4. Discussion

This study aims to explore the potential mechanism of XTSJF in the treatment of GC. 135 chemical components, namely quercetin, β-sitosterol, naringenin, nobiletin, and kaempferol, have been identified using TCMSP and the SymMap database. These main components can interact with other active elements to act on a variety of targets across the network. Among the compounds, studies have shown that quercetin, β-sitosterol, naringenin, nobiletin, and other substances have a good therapeutic effect on GC.

Quercetin, abundantly present in various edible and medicinal plants, serves as a multifunctional agent, acting as a metal chelator and ROS scavenger to safeguard gastric epithelial cells against oxidative damage.^[17,18]^ The ubiquitously prevalent β-sitosterol, a phytosterol, exhibits a spectrum of attributes encompassing anti-inflammatory, anti-cancer, and antioxidant properties.^[19,20]^ In AGS cells, β-sitosterol showcases its anticancer potential by modulating MAPK, PTEN, and Hsp90 signaling.^[21]^ Naringenin, a flavanone compound found in citrus fruits and medicinal dishes, has been extensively investigated for its anticancer virtues. Its influence spans apoptosis induction, cell cycle arrest, angiogenesis suppression, and modulation of diverse signal transduction cascades.^[22]^ Nobiletin, a flavonoid primarily found in citrus fruits, exerts anti-inflammatory effects and curtails the proliferation of various cancer cell lines. Notably, nobiletin impedes GC cell line proliferation by inducing G0-G1 phase cell cycle arrest.^[23]^ Collectively, these findings underscore the pivotal role of these components within XTSJF’s therapeutic framework in GC treatment, meriting further in-depth exploration.

In this study, a meticulous exploration of potential therapeutic targets of XTSJF in the context of GC was undertaken. An extensive assemblage of 2074 GC-related targets was curated from the OMIM and GeneCards databases. Through rigorous comparative analysis utilizing Venn diagrams, a refined set of 167 targets, signifying the intersection of GC and XTSJF-related targets, emerged as potential mediators of XTSJF’s therapeutic actions in GC. Through the establishment of a Protein-Protein Interaction network, a nucleus of pivotal targets was unveiled. Notably, TP53, MAPK3, MAPK1, STAT3, AKT1, RELA, and FOS, with particular emphasis on TP53, MAPK3, MAPK1, STAT3, and AKT1, emerged as potential core entities. TP53, acknowledged as a tumor suppressor gene, orchestrates vital regulatory roles in cell autophagy, apoptosis, cell cycle, and DNA repair.^[24]^ Mutations in TP53 can incite diverse ramifications encompassing aberrant tumor proliferation, migration, survival, and progression, subsequently impacting treatment response and prognosis.^[25]^

The MAP kinase family members, MAPK3 and MAPK1, implicated in multifarious cellular activities, including differentiation, transcriptional control, and development, displayed a significant presence.^[26,27]^ Robust expression of MAPK1 was evidenced in GC patients, further underscoring its significance in GC development.^[28]^ STAT3, a protuberant oncogene, exerts pivotal roles in cell proliferation, angiogenesis, metastasis, and immune evasion.^[29,30]^ Similarly, AKT1, a serine/threonine-specific protein kinase, assumes a cardinal role in glucose metabolism, cell division, cell migration, and survival.^[31,32]^ Importantly, AKT1’s role in GC development and growth of GC cells was substantiated.^[33]^

We employ a comprehensive approach integrating GO analysis and Kyoto Encyclopedia of Genes and Genomes (KEGG) enrichment analysis to illuminate the intricate mechanistic terrain underpinning XTSJF’s therapeutic influence in GC. Substantive GO analysis unveiled target gene enrichment predominantly within key biological processes, encompassing positive regulation of RNA polymerase II promoter transcription, negative regulation of the apoptotic process, and positive regulation of gene expression. Concurrently, KEGG enrichment analysis elucidated multifaceted intersections between XTSJF and critical signaling pathways, with a notable emphasis on the TNF, PI3K-Akt, and MAPK pathways. Among these, the TNF pathway emerges as pivotal, with TNF, a pivotal inflammatory cytokine, orchestrating a dichotomous impact on cytokine release, apoptosis, and programmed cell necrosis. Notably, TNF’s binding to receptors precipitates concurrent activation of both the caspase-8-mediated apoptotic cascade and the NF-kB-mediated inflammatory signaling axis. The intricate nexus between the PI3K/AKT pathway, key transcription factors, and pivotal signaling cascades, such as TGF-β, NFκβ, and Wnt/catenin, holds paramount significance, orchestrating outcomes spanning epithelial-mesenchymal transition, metastasis, and invasion.^[34]^ Aberrant PI3K/AKT pathway activation stands pivotal in GC development, exerting influence through the regulation of apoptotic and pro-apoptotic genes, thereby impinging upon cell survival, metastasis, and evasion of apoptosis. The MAPK signaling pathway, pivotal for diverse cellular processes including proliferation, differentiation, apoptosis, and stress response,^[26]^ is also salient within this context. CircRNA derived from the MAPK gene, like circMAPK1, has been demonstrated to suppress malignant biological behavior in GC cells, further exemplifying MAPK’s regulatory roles.^[28]^ Strikingly, the significance of MAPK1 in GC development is reinforced by its substantial expression in GC patients. This investigation illuminates the labyrinthine interactions between XTSJF and pivotal signaling pathways, augmenting our comprehension of XTSJF’s mechanism of action in GC. These findings furnish a robust foundation for further studies, unraveling the potential therapeutic avenues in the realm of GC treatment.

In light of the network pharmacological insights elucidated above, this study delves into the potential molecular mechanism underpinning XTSJF’s efficacy in GC. Utilizing a judicious approach, we identified 6 active components and 5 representative targets that form the crux of XTSJF’s therapeutic impact. To validate the predictions furnished by network pharmacology, we subjected quercetin, β-sitosterol, naringenin, nobiletin, kaempferol, and luteolin – the 6 key compounds – to molecular docking against TP53, MAPK3, MAPK1, STAT3, and AKT1 proteins, respectively. Impressively, the docking analyses affirmed strong binding interactions between the major active molecules within XTSJF and these hub targets.

The next phase of our inquiry centered on corroborating these findings through diverse databases. Remarkably, our exploration unveiled significantly elevated mRNA levels of TP53 and MAPK3 within GC tissues. Furthermore, the TP53 levels exhibited marked variations across pathological stages. Employing the cBioPortal platform, we uncovered genetic alterations within these 5 target entities in a substantial proportion of patients – 57% out of 660 cases. The corroboration of our findings with extant literature reports underscores the robustness of our analytical framework.

In essence, this investigation harnesses a comprehensive approach that amalgamates network pharmacology and molecular docking, revealing potential interactions within a multi-component, multi-target, and multi-pathway framework, which are substantiated through molecular docking analyses, enabling a deeper understanding of XTSJF’s mechanistic intricacies within the context of GC. The congruence between our findings and established literature substantiates the potency of our investigative methodology, paving the way for a nuanced comprehension of XTSJF’s clinical utility in GC treatment.

Through network pharmacology, we have identified quercetin, β-sitosterol, naringenin, epinephrine, kaempferol, and luteolin as pivotal bioactive constituents of XTSJ’s influence on GC. Nevertheless, we acknowledge that these key components might not comprehensively encapsulate XTSJ’s complete pharmacological effects. Besides, it is imperative to acknowledge the ongoing necessity for refining database accuracy and timeliness, as well as recognizing the evolving nature of web-based information technology. While recognizing the current limitations in database accuracy and the evolving landscape of information technology, we underscore the necessity of corroborative pharmacodynamic and molecular biology investigations to bolster the credibility of our findings.

## 5. Conclusions

This study offers a preliminary assessment of the pivotal constituents and potential mechanisms underscoring XTSJF’s therapeutic implications in GC, employing an integrative approach encompassing network pharmacology, molecular docking, and bioinformatics analysis. Our investigation points to quercetin, β-sitosterol, naringenin, nobiletin, kaempferol, and luteolin as the principal active components within XTSJF, while protein targets such as TP53, MAPK3, MAPK1, STAT3, and AKT1 emerge as plausible therapeutic targets for XTSJF’s impact on GC. Our findings suggest that XTSJF may harness its therapeutic potential through the modulation of critical pathways, including the PI3K-AKT signaling pathway, MAPK signaling pathway, and TNF signaling pathway. However, it is paramount to acknowledge that existing web-based information technology necessitates further refinement to ensure data accuracy and timeliness. Moreover, the significance of our results rests on the premise of rigorous experimental validation. In summary, this study adopts a multidisciplinary approach, amalgamating network pharmacology, molecular docking, and bioinformatics analysis, to offer a preliminary exploration of XTSJF’s therapeutic role in GC. As we navigate the evolving landscape of data technology, and recognize the essentiality of experimental confirmation, our study paves the way for a more comprehensive understanding of XTSJF’s potential efficacy in the context of GC treatment.

## Author contributions

**Data curation:** Xinxin Xu, Shuying Zeng.

**Formal analysis:** Xinxin Xu.

**Investigation:** Xinxin Xu.

**Supervision:** Zhihong Yu.
